# Japanese Subgroup Analyses from EMERGE and ENGAGE, Phase 3 Clinical Trials of Aducanumab in Patients with Early Alzheimer’s Disease

**DOI:** 10.14283/jpad.2024.106

**Published:** 2024-07-27

**Authors:** Yasuo Toda, T. Iwatsubo, Y. Nakamura, N. Matsuda, M. Miyata, M. Jin, T. Chen, K. Kuribayashi, Y. Tian, R. Hughes, J. Yamamoto, K. K. Muralidharan, C. Rubel, R. M. Hutchison, S. Budd Haeberlein

**Affiliations:** 1 Biogen Japan Ltd., 1-4-1 Nihonbashi, Chuo-ku, Tokyo, 103-0027 Japan; 2 Unit for Early and Exploratory Clinical Development, The University of Tokyo Hospital, Bunkyo-ku, Tokyo, 113-8655 Japan; 3 Department of Neuropathology, Graduate School of Medicine, The University of Tokyo, Bunkyo-ku, Tokyo, 113-0033 Japan; 4 Department of Neuropsychiatry, Faculty of Medicine, Kagawa University, Kita-gun, Kagawa, 761-0793 Japan; 5 Biogen Inc., 225 Binney St, Cambridge, MA 02142 USA; 6 Enigma Biomedical Group USA, Knoxville, TN 37922 USA

**Keywords:** Aducanumab, early Alzheimer’s disease, biomarker, amyloid beta, plasma p-tau181, PK, immunogenicity, Japan, subgroup analyses

## Abstract

**Background:**

Global prevalence and incidence of dementia continue to rise at a rapid rate. There is a need for new Alzheimer’s disease (AD) treatments globally. Aducanumab is a human monoclonal antibody that selectively targets aggregated soluble amyloid beta oligomers and insoluble amyloid beta fibrils. In June 2021, aducanumab was approved by the US Food and Drug Administration for the treatment of AD under the accelerated approval pathway.

**Objectives:**

We evaluated the efficacy, safety, biomarker and pharmacokinetics (PK) of aducanumab in Japanese subgroups in EMERGE and ENGAGE studies.

**Design:**

EMERGE and ENGAGE were two randomized, double-blind, placebo-controlled, global, phase 3 studies of aducanumab in patients with early AD (mild cognitive impairment due to AD or mild AD dementia).

**Setting:**

These studies involved 348 sites in 20 countries. PARTICIPANTS: Participants enrolled in Japan included 121 (7.4% of total 1638 in EMERGE) and 100 (6.1% of total 1647 in ENGAGE) patients (aged 50–85 years with confirmed amyloid pathology) who met clinical criteria for mild cognitive impairment due to AD or mild AD dementia.

**Intervention:**

Participants were randomly assigned 1:1:1 to receive aducanumab low dose (3 or 6 mg/kg target dose), high dose (6 or 10 mg/kg target dose) or placebo via IV infusion once every 4 weeks over 76 weeks.

**Measurements:**

The primary outcome measure was change from baseline to Week 78 on the Clinical Dementia Rating Sum of Boxes (CDR-SB), an integrated scale that assesses both function and cognition. Other measures included safety assessments; secondary and tertiary clinical outcomes that assessed cognition, function, and behavior; biomarker endpoints (amyloid PET and plasma p-tau181); serum PK profiles and immunogenicity.

**Results:**

Results from the Japanese subgroup analyses were generally consistent with those of the overall study population across endpoints, while a lower mean body weight (kg) and a smaller proportion of ApoE ε4 carriers were observed in the Japanese subgroup population. A treatment effect was observed in favor of aducanumab on the primary and secondary efficacy endpoints at Week 78 in EMERGE, but not ENGAGE. The incidence and type of adverse events in the Japanese subgroups were generally comparable to those observed in the overall study population; amyloid related imaging abnormalities (ARIA) were common treatment-related adverse events that appeared to be related to the aducanumab dose. ARIA incidence was generally lower in the Japanese subgroup compared with the overall population. Consistent with the overall data set, a robust dose-dependent decrease in amyloid beta levels as assessed with amyloid-PET and plasma p-tau181 was observed. Serum PK profiles and immunogenicity of aducanumab in Japanese population were consistent with the non-Japanese population.

**Conclusion:**

Efficacy, safety, biomarker, and PK profiles of aducanumab were consistent between the Japanese subgroup and the overall population. A positive treatment effect of aducanumab on efficacy endpoints was observed in EMERGE, but not in ENGAGE.

**Electronic Supplementary Material:**

Supplementary material is available in the online version of this article at 10.14283/jpad.2024.106.

## Introduction

**G**lobal prevalence and incidence of dementia continue to rise at a rapid rate. It is estimated that approximately 50 million people worldwide are affected by dementia, a number that is projected to triple by 2050 (1). Among the multiple etiologies, Alzheimer’s disease (AD) is the most common cause of dementia (2). These statistics also apply to Japan, one of the largest and fastest aging countries (3). The age-standardized number of Japanese individuals with AD and other dementia was approximately 3.5 million in 2016, which was a 17.8% increase from 1990 (4) and is projected to affect 20% (7 million) of older adults in Japan by 2025 (5). With no cure available to date, AD remains a burdensome disease in the US (2) as well as in Japan. For instance, ¥1.91 trillion ($14.8 billion USD) were spent in 2014 on dementia-related healthcare (6). Physical and emotional burdens also affect Japanese caregivers of patients including AD; caregivers of patients with AD or dementia in Japan experienced decreases in quality of life and work productivity and an increase in depression and anxiety compared with noncaregivers (7). Slowing of disease progression in AD by a disease modifying therapy is expected to enable patients with early AD to spend more time in earlier stages of AD and in the community, improve quality of life for patients and caregivers, and reduce community and residential care costs (8).

AD is a progressive disorder that is characterized by pathological deposits of amyloid beta (Aβ) and tau in the brain (2, 9). Considering a global need for new AD treatments (10), significant efforts have been made to develop therapies including anti-Aβ monoclonal antibody treatments that target underlying AD pathologies (11–15). Aducanumab is a human monoclonal antibody that selectively targets Aβ aggregates (14, 16, 17). EMERGE (NCT02484547) and ENGAGE (NCT02477800) were global Phase 3, multicenter, randomized, double-blind, placebo-controlled, parallel-group clinical trials that evaluated the efficacy and safety of aducanumab in patients with early AD with confirmed amyloid pathology (18–20).

Final analyses were conducted on the Phase 3 EMERGE and ENGAGE data via the prespecified analysis plan and the final results have been reported (20). Briefly, EMERGE, but not ENGAGE, met its primary endpoint of reduced clinical decline with a significant treatment effect of high-dose aducanumab vs placebo that was (8) consistent across prespecified primary and secondary clinical endpoints (21). Furthermore, EMERGE and ENGAGE demonstrated a dose- and time-dependent reduction in biomarkers of AD pathophysiology (20). Based on the lowering of brain Aβ plaque levels assessed with amyloid-PET observed in clinical trials of aducanumab, the U.S. Food and Drug Administration (FDA) provided the approval to aducanumab on June 07, 2021 for the treatment of AD under accelerated pathway (16).

It remains unclear whether race or ethnicity contributes to AD prevalence (22). However, data from Japanese Alzheimer’s Disease Neuroimaging Initiative (J-ADNI) suggest that there may be racial or ethnic differences that contribute to prevalence and rates of disease progression. The J-ADNI study showed Japanese participants had a disease progression similar to the North American ADNI in the late stage of mild cognitive impairment, but it was milder than North American ADNI in mild AD participants (23). In addition, J-ADNI has a lower prevalence of apolipoprotein E (ApoE) ε4 carrier status, a risk factor for AD, than North American ADNI as shown in previous report (23, 24). Given that participants in North America are often overrepresented in AD trials (25, 26), it is important to evaluate the efficacy and safety of AD treatments in the Japanese population who are also affected by the human and economic burden of AD. A prespecified subgroup analysis was conducted in order to evaluate the efficacy and safety of aducanumab in Japanese participants in EMERGE and ENGAGE relative to the overall study populations.

## Methods

### Participants

Inclusion criteria for EMERGE and ENGAGE are listed (20). Briefly, EMERGE and ENGAGE included patients with early AD who 1) met clinical criteria for either mild cognitive impairment (MCI) due to AD (27) or mild AD dementia (28), 2) were aged 50 to 85 years, 3) had a Clinical Dementia Rating (CDR)-Global score of 0.5, 4) had a Repeatable Battery for the Assessment of Neuropsychological Status (RBANS) delayed memory index score ≤85, and 5) had a Mini-Mental State Examination (MMSE) score between 24 and 30 (inclusive) (18–20). Participants were confirmed to be amyloid positive via amyloid positron emission tomography (PET; central visual read). Participants in Japan were enrolled across 27 sites (EMERGE) and 18 sites (ENGAGE).

### Clinical study design

Details have been previously published (20). Briefly, randomization was stratified by study site and by apolipoprotein E (ApoE) ε4 carrier status (carrier vs noncarrier). Participants were randomized in a 1:1:1 ratio to receive low-dose aducanumab (3 mg/kg [ApoE ε4+] or 6 mg/kg [ApoE ε4−]), high-dose aducanumab (6 mg/kg [ApoE ε4+] or 10 mg/kg [ApoE ε4−]), or placebo via intravenous infusion every 4 weeks. The implementation of protocol version 4 (PV4; approved on March 24, 2017) allowed ApoE ε4 carriers in the high-dose group to receive 10 mg/kg of aducanumab, which was an increase from 6 mg/kg pre-PV4. The treatment period for the Japanese study sites was from June 9, 2016, to July 31, 2019 (final follow-up visit) in EMERGE and from September 1, 2016, to July 31, 2019 (final follow-up visit) in ENGAGE.

### Efficacy assessments

The efficacy outcomes included the primary endpoint (change from baseline in CDR-Sum of Boxes [SB] at Week 78) and secondary endpoints (change from baseline in MMSE, Alzheimer Disease Assessment Scale-Cognitive Subscale-13 items [ADAS-Cog13], and Activities of Daily Living Inventory-Mild Cognitive Impairment version [ADCS-ADL-MCI] scores at Week 78) (20).

### Safety assessments

A description of the safety assessments in EMERGE and ENGAGE, including methodology related to the monitoring, analyses, and management of amyloid related imaging abnormality (ARIA), have been reported (20, 29). Briefly, the safety and tolerability of aducanumab were assessed via (1) monitoring incidence of adverse events (AEs) and serious adverse events (SAEs); (2) assessing brain magnetic resonance imaging (MRI) findings (e.g., the incidence of ARIA-edema [E] and ARIA-hemorrhage [H] comprised of microhemorrhages, macrohemorrhages, or superficial siderosis); (3) monitoring clinically significant abnormalities and changes in vital signs, (4) tracking Columbia Suicide Severity Rating Scale results, and (5) monitoring incidence of anti-aducanumab antibodies in serum throughout the study period.

### Biomarkers, pharmacokinetics and immunogenicity

Cerebral amyloid plaque content was measured by amyloid PET imaging for eligibility and as part of a longitudinal biomarker substudy, as previously described (20, 30). Images were collected at baseline, Week 26 and Week 78. Multiple radioligands for Aβ PET have been approved for the detection of amyloid. Globally, EMERGE and ENGAGE trial sites used the 18F-florbetapir radioligand (31) for substudy imaging, however due to the its limited geographic availability in Japan at the start of the studies, the 18F-flutemetamol radioligand (32) was also included. Both tracers have been approved for the detection of amyloid in Japan. The Centiloid Scale approach was used to transform Aβ PET composite standardized uptake value ratio (SUVR) derived from each PET radioligand to a common scale to enable pooling of results (33). The formulas used were: 100×((SUVR-1.0124))/0.4339 for 18F-florbetapir and 100×((SUVR-0.9593))/0.6170 for 18F-flutemetamol.

The effects of aducanumab treatment on plasma p-tau181 levels were analyzed using the Quanterix Simoa p-tau181 Advantage V2 kit at Frontage Laboratories’ (Exton, PA) CLIA laboratory. Data were captured by the Quanterix Simoa HD-X Analyzer as previously described (20,30). Serum PK profiles and immunogenicity of aducanumab were measured throughout the study using a validated assay.

### Oversight

EMERGE and ENGAGE were carried out in accordance with the Code of Ethics of the World Medical Association (Declaration of Helsinki) and the International Council for Harmonization Good Clinical Practice guideline (ICH-GCP). The studies were approved by ethics committees or institutional review boards at each participating site in Japan. All patients provided written informed consent. An independent data monitoring committee reviewed ongoing safety and tolerability data throughout the course of the studies and reviewed futility analysis results.

### Statistical analyses

Primary analyses on the primary, secondary, and amyloid PET endpoints for the Japanese subgroup were performed following the same prespecified mixed model for repeated measures (MMRM) used for the overall population (19), except for the exclusion of region as a covariate. Forest plots of the efficacy results were presented to evaluate the consistency of the Japanese efficacy results with those of the overall population. Incidence was calculated by treatment group for AEs and SAEs in the Japanese subgroup. The statistical software, SAS®, was used for all summaries and analyses.

### Availability of data and materials

While the data described in this article are not publicly available, the authors and Biogen are supportive of data sharing. Biogen has established processes to share protocols, clinical study reports, study-level data, and de-identified patient-level data. These data and materials will be made available to qualified scientific researchers in support of the objective(s) in their approved, methodologically sound research proposal. Proposals should be submitted through Vivli (https://vivli.org). To gain access, data requestors will need to sign a data sharing agreement. For general inquiries, please contact datasharing@biogen.com. Biogen’s data-sharing policies and processes are detailed on the website www.biogentrialtransparency.com

## Results

### Disposition and baseline characteristics

**Figure 1:**
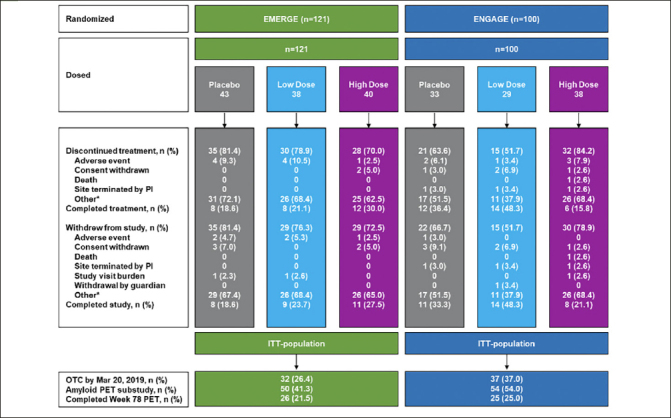
Flow chart of participant disposition for the EMERGE and ENGAGE Japanese subgroup analyses Abbreviation: ITT, intent-to-treat; OTC, opportunity to complete; PI, principal investigator. *This category contains discontinuation and withdrawal due to termination of the studies.

A total of 121 (7.4% of total 1638 in EMERGE) and 100 (6.1% of total 1647 in ENGAGE) participants enrolled in Japan were randomized and received ≥1 dose of treatment during the placebo-controlled period. Participants who completed study were 23% (n=28) in EMERGE and 33% (n=33) in ENGAGE (Fig. 1). PV4 was approved on March 24, 2017 and implemented across global sites over approximately 18 months (20). In general, the enrollment for ENGAGE occurred ahead of that for EMERGE (Supplemental Fig. 1). Baseline demographics and disease characteristics were generally balanced across EMERGE and ENGAGE and across treatment groups in the Japanese subgroup (Table 1). Subgroup participants were, by definition, participants enrolled in Japanese sites. All subgroup participants identified as Japanese for race. Baseline demographics and disease characteristics, including CDR-SB scores, were generally consistent between the overall population and the Japanese subgroup (Supplemental Table 1). Differences included a lower mean body weight (kg) and a smaller proportion of ApoE ε4 carriers in the Japanese subgroup compared with the overall population (Supplemental Table 1).

**Table 1:** Baseline demographics and disease characteristics of the Japanese subgroup

	**EMERGE**			**ENGAGE**		
	**Placebo (n=43)**	**Low dose (n=38)**	**High dose (n=40)**	**Placebo (n=33)**	**Low dose (n=29)**	**High dose (n=38)**
Age, mean ± SD, years	71.6±8.12	70.9±8.15	70.8±8.86	70.4±7.95	73.4±6.93	72.2±6.50
Female, n (%)	23 (53.5)	19 (50.0)	19 (47.5)	16 (48.5)	18 (62.1)	23 (60.5)
Race						
Japanese	43 (100)	38 (100)	40 (100)	33 (100)	29 (100)	38 (100)
Weight, mean ± SD, kg	54.98±10.183	55.56±9.859	55.67±8.723	53.26±9.885	51.96±10.356	55.00±7.856
Years of education, mean ± SD	12.7±2.91	13.8±2.43	13.9±2.64	13.7±2.57	12.9±2.15	13.7±2.18
AD medications used, n (%)	27 (62.8)	23 (60.5)	26 (65.0)	22 (66.7)	19 (65.5)	20 (52.6)
ApoE ε4, n (%)						
Carriers	23 (53.5)	24 (63.2)	25 (62.5)	21 (63.6)	22 (75.9)	24 (63.2)
Homozygote ε4	4 (9.3)	3 (7.9)	4 (10.0)	5 (15.2)	0	6 (15.8)
Heterozygote ε4	19 (44.2)	21 (55.3)	21 (52.5)	16 (48.5)	22 (75.9)	18 (47.4)
Noncarriers	20 (46.5)	14 (36.8)	15 (37.5)	12 (36.4)	7 (24.1)	14 (36.8)
Clinical stage, n (%)						
MCI due to AD	33 (76.7)	31 (81.6)	23 (57.5)	28 (84.8)	22 (75.9)	32 (84.2)
Mild AD	10 (23.3)	7 (18.4)	17 (42.5)	5 (15.2)	7 (24.1)	6 (15.8)
RBANS delayed memory score, mean ± SD	59.7±16.05	57.0±13.74	55.1±13.59	54.1±12.42	51.4±11.37	59.3±15.22
MMSE, mean ± SD	25.9±1.72	26.2±1.87	26.4±1.46	26.2±1.86	25.6±1.76	26.6±1.60
CDR global score 0.5, n (%)	43 (100)	38 (100)	40 (100)	33 (100)	29 (100)	38 (100)
CDR-SB, mean ± SD	2.15±0.842	2.34±1.053	2.53±1.224	2.21±0.829	2.34±1.053	1.83±1.028
ADAS-Cog13, mean ± SD	22.3±4.86	21.9±4.94	21.5±7.32	22.7±6.58	24.6±6.47	20.9±5.83
ADCS-ADL-MCI, mean ± SD	40.4±5.32	40.9±5.64	39.2±7.66	41.1±5.62	41.8±6.16	42.7±4.98
Amyloid PET substudy population, n	19	15	16	18	14	22
Amyloid PET centiloid values, mean ± SD	74.8±26.19	71.6±25.68	71.0±35.78	82.0±2.95	78.1±34.92	92.0±29.27
plasma pTau181 analysis population, n	8	7	11	12	14	8
plasma pTau181, mean ± SD, pg/ml	3.045±0.5726	2.954±0.8620	3.185±0.9766	3.568±0.9081	3.054±1.1165	3.118±1.4215

AD, Alzheimer’s disease; ADAS-Cog13, Alzheimer’s Disease Assessment Scale-Cognitive Subscale (13 items); ADCS-ADL-MCI, Alzheimer’s Disease Cooperative Study–Activities of Daily Living Inventory (Mild Cognitive Impairment version); ApoE, apolipoprotein E; CDR, Clinical Dementia Rating; CDR-SB, CDR-Sum of Boxes; MCI, mild cognitive impairment; MMSE, Mini-Mental State Examination; PET, positron emission tomography; RBANS, Repeatable Battery for the Assessment of Neuropsychological Status; SD, standard deviation

### Clinical efficacy

**Figure 2:**
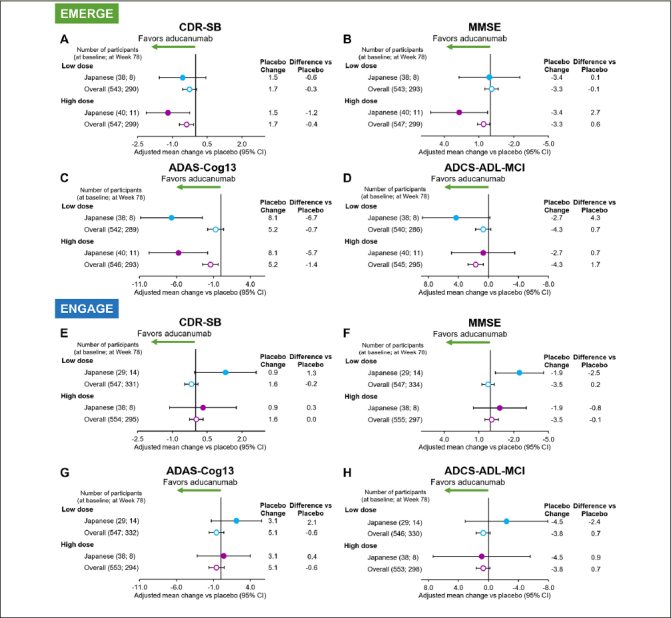
Forest plots of adjusted mean change from baseline vs placebo at Week 78 on CDR-SB, MMSE, ADAS-Cog13, and ADCS-ADL-MCI in EMERGE (A-D) and ENGAGE (E-H) ADAS-Cog13, Alzheimer’s Disease Assessment Scale-Cognitive Subscale (13 items); ADCS-ADL-MCI, Alzheimer’s Disease Cooperative Study-Activities of Daily Living Inventory (Mild Cognitive Impairment version); CDR-SB, Clinical Dementia Rating CDR-Sum of Boxes; CI, confidence interval; MMSE, Mini-Mental State Examination.

The number of participants analyzed at Week 26/50/78 were 119/79/27 in EMERGE and 95/78/33 in ENGAGE respectively. Consistent with the overall population, a positive treatment effect of high-dose aducanumab compared with placebo was observed on the primary endpoint, change from baseline in CDR-SB score at Week 78, in the EMERGE Japanese subgroup (−1.19 vs placebo [95% CI, −2.120 to −0.257]; Fig. 2A). A numerical advantage of aducanumab treatment on the primary endpoint was observed in the low-dose group (−0.56 vs placebo [95% CI, −1.567 to 0.442]).

A positive treatment effect of high-dose aducanumab was also observed across the secondary endpoints in the EMERGE Japanese subgroup. High-dose aducanumab showed a numerical advantage in favor of aducanumab on change from baseline to Week 78 in MMSE (2.7 vs placebo [95% CI, 0.32 to 5.14]; Fig. 2B), ADAS-Cog13 (−5.731 vs placebo [95% CI, −9.6971 to −1.7657]; Fig. 2C), and ADCS-ADL-MCI (0.7 vs placebo [95% CI, −3.53 to 4.91]; Fig. 2D). Overall, the 95% CI of the low- and high-dose aducanumab arms were wide but contained point estimates for the EMERGE overall population in 3 out of 4 clinical endpoints, suggesting general consistency.

In the previously reported ENGAGE overall results (20), ENGAGE did not meet its primary or secondary endpoints. Consistent with this observation, no treatment effect was observed on the ENGAGE primary or secondary endpoints in the Japanese subgroup compared with placebo (Fig. 2E–H).

### Amyloid PET imaging and plasma p-tau181 results

**Figure 3:**
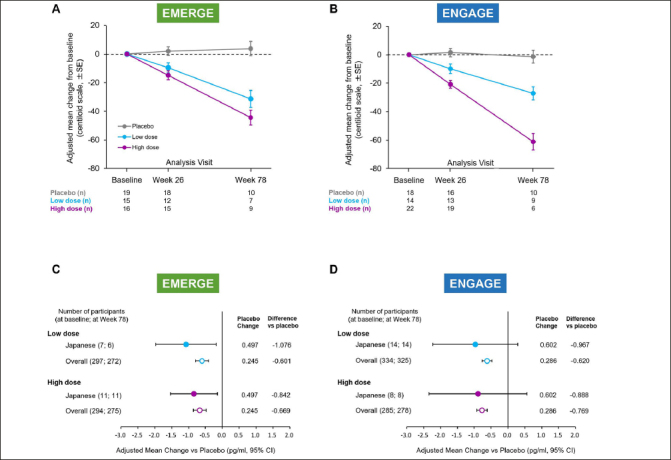
Change from baseline of amyloid PET (centiloid units) and plasma p-tau181 level at Week 78 Mean change from baseline of amyloid PET centiloid values in amyloid PET substudies of EMERGE (A) and ENGAGE (B). Error bars denote standard error. Mean change vs placebo of plasma p-tau181 levels (pg/ml) at Week 78 in the plasma p-tau181 analysis populations of EMERGE (C) and ENGAGE (D). PET, positron emission tomography; CI, confidence interval.

Amyloid PET images were collected longitudinally in a cohort of the Japanese subgroup as described in Methods (The number of data available at baseline/Week26/Week78 were 50/45/26 in EMERGE and 54/48/25 in ENGAGE respectively.). Consistent with the overall population (20), a dose- and time-dependent reduction in brain Aβ plaque levels was observed in each study compared with placebo (Fig. 3A, B). At Week 78, high-dose aducanumab decreased brain Aβ plaque levels (centiloid units) by −48.2 (95% CI, −63.1 to −33.2) in EMERGE (n=9) and −59.9 (95% CI, −75.0 to −44.7) in ENGAGE (n=6). In the low-dose arm, the difference from placebo in mean centiloid units at Week 78 was −35.1 (95% CI, −51.6 to −18.5) in EMERGE (n=7) and −25.8 (95% CI, −38.9 to −12.7) in ENGAGE (n=9).

Plasma p-tau181 was assayed in available plasma samples from participants in the Japanese subgroup as part of the batched analysis of the overall study samples (EMERGE: n=18; ENGAGE: n=22). Consistent with the overall population (20), compared to placebo, reductions in plasma p-tau181 levels were observed at Week 78 in both high-and low-dose aducanumab arms in EMERGE (Fig. 3C) and ENGAGE (Fig. 3D). At Week 78, the difference in adjusted mean change from baseline between placebo and high-dose aducanumab was −0.842 (95% CI, −1.540 to −0.144) for EMERGE and −0.888 (95% CI, −2.342 to 0.566) for ENGAGE. In the low dose arm, the difference was −1.076 (95% CI, −1.974 to −0.178) for EMERGE and −0.967 (95% CI, −2.222 to 0.288) for ENGAGE.

### Exposure to study treatment

Due to the termination of the studies on March 21, 2019, majority of the ITT population in the Japanese subgroups did not receive the full 78-week treatment. The mean study treatment duration was 58.89 weeks in EMERGE and 61.33 weeks in ENGAGE. Thirty-two participants (26.4%) in EMERGE and 37 participants (37.0%) in ENGAGE had the opportunity to complete (OTC) the Week 78 assessments prior to the futility announcement. In these participants, the mean (SD) cumulative dose received in the low-dose group was 51.8 mg/kg (21.58 mg/kg) in EMERGE (n=9) and 63.3 mg/kg (25.75 mg/kg) in ENGAGE (n=15), and in the high-dose group, 126.8 mg/kg (50.51 mg/kg) in EMERGE (n=13) and 119.4 mg/kg (47.28 mg/kg) in ENGAGE (n=10). The latter difference was a direct result of a greater mean (SD) number of 10 mg/kg doses received in EMERGE than in ENGAGE (10.5 [4.81] and 9.4 [4.77], respectively) (Supplemental Table 2).

The serum PK profiles of aducanumab were similar between the non-Japanese and the Japanese subgroup. As expected, mean trough serum concentrations of aducanumab generally increased in a dose-proportional manner from 3 mg/kg to 10 mg/kg after Week 24. Furthermore, in all aducanumab dose groups, serum aducanumab concentration reached a steady-state within the treatment period in both studies (Supplemental Fig. 2).

### Safety

Safety findings in the Japanese subgroup were generally consistent with those of the overall population. The number of participants who experienced any AEs was comparable between the placebo and aducanumab groups (low- dose and high- dose) in each study (Table 2). The majority of participants experienced AEs with a maximum severity of mild (EMERGE, 55.0%–67.4%; ENGAGE, 47.4%–55.2%) or moderate (EMERGE, 16.3%–30.0%; ENGAGE, 24.1%–27.3%), and fewer participants experienced severe AEs (EMERGE, 2.5%–10.5%; ENGAGE, 0%–5.3%). The incidence of SAEs is listed in Table 2. There was no fatal AE that, in the opinion of the investigator, were considered treatment related.

**Table 2:** Summary of adverse events of the Japanese subgroup

	**EMERGE**			**ENGAGE**		
**n (%)**	**Placebo (n=43)**	**Low dose (n=38)**	**High dose (n=40)**	**Placebo (n=33)**	**Low dose (n=29)**	**High dose (n=38)**
Patients with any AE	38 (88.4)	35 (92.1)	35 (87.5)	25 (75.8)	23 (79.3)	30 (78.9)
Patients with any SAE	8 (18.6)	7 (18.4)	2 (5.0)	5 (15.2)	3 (10.3)	6 (15.8)
Nasopharyngitis	13 (30.2)	7 (18.4)	8 (20.0)	4 (12.1)	7 (24.1)	9 (23.7)
ARIA-H	1 (2.3)	5 (13.2)	8 (20.0)	3 (9.1)	2 (6.9)	5 (13.2)
ARIA-E	0	5 (13.2)	8 (20.0)	1 (3.0)	2 (6.9)	5 (13.2)
Contusion	3 (7.0)	0	2 (5.0)	1 (3.0)	3 (10.3)	4 (10.5)
Fall	5 (11.6)	1 (2.6)	6 (15.0)	2 (6.1)	4 (13.8)	3 (7.9)
Superficial siderosis of central nervous system	0	2 (5.3)	2 (5.0)	0	1 (3.4)	3 (7.9)
Deaths	0	0	0	0	0	1 (2.6)*

*Was determined not related to study drug as assessed by the investigator. AE, adverse events, ARIA, amyloid related imaging abnormality; ARIA-E; ARIA-edema/effusion; ARIA-H, ARIA-microhaemorrhages and haemosiderin deposits, SAE, severe adverse events.

The effects of long-term exposure to aducanumab on immunogenicity were low in Japanese participants. Specifically, in EMERGE, the incidence of treatment-emergent anti-aducanumab antibodies was low (placebo, 0/43 [0%]; low- dose, 1/37 [2.7%]; high- dose, 0/40 [0%]) and transient. Similarly, in ENGAGE, no treatment-emergent anti-aducanumab antibody incidence was observed. Common AEs (≥10% incidence) in EMERGE and ENGAGE that occurred more frequently in the aducanumab-treated patients were ARIA-E, ARIA-H, and fall. Compared with the overall population, the incidence of ARIA was lower in the Japanese subgroup (20, 29) and the incidence of ARIA-E was highest in the high-dose aducanumab group (Supplemental Table 3). Participants in the high-dose groups or who were ApoE ε4 carriers were generally observed to have a higher incidence of ARIA-E. Furthermore, ARIA-E was mostly asymptomatic (92.3% in EMERGE and 71.4% in ENGAGE).

## Discussion

The present subgroup analyses are the first to detail the clinical efficacy and safety of the anti-Aβ monoclonal antibody aducanumab in Japanese patients with early AD. Results were consistent with those of the EMERGE and ENGAGE overall population recruited in 20 countries (20). Specifically, a treatment effect in favor of high-dose aducanumab was observed across primary and secondary endpoints in EMERGE, whereas in ENGAGE, a treatment effect favoring aducanumab was not observed. Importantly, aducanumab treatment resulted in a robust dose- and time-dependent reduction in brain amyloid plaque burden in the Japanese subgroup in a manner comparable to results in the overall population in both studies.

Baseline demographics and disease characteristics of the Japanese subgroup were generally comparable to that of the overall population (20); however, a few expected differences were observed (Supplemental Table 1). Specifically, a smaller proportion of patients were ApoE ε4 carriers in the Japanese subgroups than in the overall population, consistent with previous reports (23, 24), while the difference in ApoE ε4 frequency in the present study requires cautious interpretation due to low sample size for each ApoE ε4 group (2 to 9 patients per group). In addition, the average body weight of the Japanese subgroup was lower than that of the overall population, in agreement with previous reports (26, 34–36). Still it is unlikely that these differences in baseline characteristics had an significant impact on the study results, considering the Japanese subgroup was generally consistent with the overall population in terms of baseline clinical scores.

Partial discordance was observed within the EMERGE and ENGAGE Japanese subgroup with respect to aducanumab clinical efficacy results; this partial discordance occurred in a similar manner in the overall population. Further context for this discordance and other potential contributing factors in the overall population are evaluated (21) and can be speculated similarly in the Japanese subgroup results.

As briefly discussed, one of the possible factors for the partial discordance seen between EMERGE and ENGAGE results in the overall data set was a difference in the proportion of patients who had full access to the 10 mg/kg dosing regimen; this discrepancy was due to the timing of PV4 implementation in EMERGE and ENGAGE, which allowed ApoE ε4 carriers to titrate up to 10 mg/kg aducanumab from 6 mg/kg (20). This difference in access to full dosing was also true for the Japanese subgroup (Supplemental Fig. 1). Indeed, the Japanese EMERGE high-dose group received a greater cumulative dose of aducanumab (average cumulative dose, 126.8 mg/kg) relative to the ENGAGE high-dose group (average cumulative dose, 119.4 mg/kg); this group also received an increased number of possible 10 mg/kg doses to full 14-dose regimen of 10 mg/kg (Supplemental Table 2). Therefore, differences in cumulative dose may also have contributed to the partial discordance in the EMERGE and ENGAGE results in the Japanese subgroups. However, the causes remain undetermined.

Consistent with results observed in the overall population, a dose- and time- dependent effect of aducanumab on brain Aβ plaque levels was observed in EMERGE and ENGAGE. However, in contrast to the overall population (20), reductions in amyloid PET (centiloid units) at Week 78 were numerically greater in the ENGAGE high-dose group (−59.9 CL) compared with the EMERGE high-dose group (−48.2 CL) Fig. 3A and 3B). This may be due to 1) a higher baseline amyloid in ENGAGE (92.0 CL) than EMERGE (71.0 CL) allowing for a greater degree of clearance amyloid to be achieved from baseline, and/or 2) the limited number of patients in the high-dose arm in the Japanese amyloid PET substudy (EMERGE, n=9; ENGAGE, n=6 at Week 78). Note that even though ENGAGE had more amyloid removal, the final amyloid load at Week 78 was still higher than EMERGE (ENGAGE 30.8 CL; EMERGE 26.6 CL). Whether these differences contributed to the partial discordance in aducanumab-induced Aβ reduction in the Japanese subgroup cannot be determined. In plasma p-tau181 substudy, consistent with overall data, aducanumab decreased plasma p-tau181 at Week 78 compared to placebo in both high- and low-dose in EMERGE and ENGAGE. Changes of plasma p-tau181 in the placebo arm of both studies are numerically higher in Japanese population compared to overall. Given the small sample size in the Japanese population in this study, confirmation of the longitudinal dynamics of plasma p-tau181 specifically in the Japanese population will be important in future studies.

Aducanumab was well tolerated, and the safety profile of the Japanese subgroup was generally comparable to that of the overall population (20). Specifically, the incidence of ARIA-E was highest in the high-dose aducanumab arms. Similar findings were observed previously in other studies of amyloid-targeting monoclonal antibodies (12, 15, 37–40). Compared with the overall EMERGE and ENGAGE high-dose groups, the incidence of ARIA-E was lower in the Japanese EMERGE and ENGAGE high-dose groups. One possible factor is the smaller percentage of ApoE ε4 carriers in the Japanese subgroup compared with the overall population. ARIA-E cases were more commonly observed in ApoE ε4 carriers than noncarriers (14, 15, 20, 40). Therefore, it is possible that the lower ApoE ε4 carriage rate in the Japanese subgroup may explain, at least in part, the lower incidence of ARIA-E in the Japanese subgroup vs the overall population. However, the incidence of ARIA in Japanese ApoE ε4 carriers (EMERGE, 20.0% [5 of 25 carriers]; ENGAGE, 20.8% [5 of 24 carriers]; Supplemental Table 3) is less than in ApoE ε4 carriers in the overall population (EMERGE, 43.1% [156 of 362 carriers]; ENGAGE, 42.1% [159 of 378 carriers]) (20), indicating there may be additional factors.

In summary, the results from the Japanese subgroups are generally consistent with the overall EMERGE and ENGAGE trial results. Importantly, the present findings suggest that high-dose aducanumab decreased brain Aβ plaque burden with an acceptable safety profile in Japanese patients. However, one of the limitations of the Japanese subgroup study was overall low sample sizes at Week 78 due to the early termination of EMERGE and ENGAGE (EMERGE, n=32 out of 121 [26.4% had opportunity to complete Week 78 assessments by the futility announcement]; ENGAGE, n=37 out of 100 [37.0% had opportunity to complete Week 78 assessments by the futility announcement]), which were largely due to the early termination of EMERGE and ENGAGE; therefore, results should be interpreted with caution. These data may serve as a foundation for fulfilling the global need for AD treatments and interventions, particularly in Japan and the larger region of Asia.

## Electronic supplementary material

Supplementary material, approximately 327 KB.

## Notes

*Disclosures:* YN did not receive any fees or special compensation from Biogen in return for authoring this publication. TC, YT, RH, KKM, CR and RMH are employees and shareholders of Biogen Inc. YT, MM, MJ, KK and JY are employees of Biogen Japan Ltd and shareholders of Biogen Inc. NM was an employee of Biogen Japan Ltd at the time of this study and has since left the company. SBH was an employee of Biogen Inc at the time of this study and has since left the company and is an employee of Enigma Biomedical Group USA.

## Notes

*Ethical standard:* The study was conducted in accordance with the Declaration of Helsinki and the International Council for Harmonization Good Clinical Practice guideline and was approved by ethics committees or institutional review boards at each site. All patients provided written informed consent. An independent data monitoring committee was responsible for monitoring the conduct of the trials, assessing routine safety, and reviewing futility analysis results.
