# Supplementary material for: Japanese Subgroup Analyses from EMERGE and ENGAGE, Phase 3 Clinical Trials of Aducanumab in Patients with Early Alzheimer’s Disease
Source: J Prev Alzheimers Dis. 2024 Jul 27;11(5):1260–9. doi: 10.14283/jpad.2024.106 (PMC11436400; doi:10.14283/jpad.2024.106)
Supplement: Supplementary file 1 — Supplementary material, approximately 327 KB. [file 42414_2024_106_MOESM1_ESM.docx]

**Supplemental Table 1. Baseline demographic and disease characteristics differences between the Japanese subgroup and the overall population**

|  | **EMERGE** | | | **ENGAGE** | | |
| --- | --- | --- | --- | --- | --- | --- |
| **Japanese** | **Placebo**  **(n=43)** | **Low dose**  **(n=38)** | **High dose**  **(n=40)** | **Placebo**  **(n=33)** | **Low dose**  **(n=29)** | **High dose**  **(n=38)** |
| CDR-SB, mean ± SD | 2.15±0.842 | 2.34±1.053 | 2.53±1.224 | 2.21±0.829 | 2.34±1.053 | 1.83±1.028 |
| Weight, mean ± SD, kg | 54.98  ±10.183 | 55.56  ±9.859 | 55.67  ±8.723 | 53.26  ±9.885 | 51.96  ±10.356 | 55.00  ±7.856 |
| ApoE ε4, n (%) |  |  |  |  |  |  |
| Carriers | 23 (53.5) | 24 (63.2) | 25 (62.5) | 21 (63.6) | 22 (75.9) | 24 (63.2) |
| Noncarriers | 20 (46.5) | 14 (36.8) | 15 (37.5) | 12 (36.4) | 7 (24.1) | 14 (36.8) |
|  | **EMERGE** | | | **ENGAGE** | | |
| **Overall** | **Placebo**  **(n=548)** | **Low dose**  **(n=543)** | **High dose**  **(n=547)** | **Placebo**  **(n=545)** | **Low dose**  **(n=547)** | **High dose**  **(n=555)** |
| CDR-SB, mean ± SD* | 2.47±1.00 | 2.46±1.01 | 2.51±1.05 | 2.40±1.01 | 2.43±1.01 | 2.40±1.01 |
| Weight, mean ± SD, kg^†^ | 71.46  ±15.373 | 73.01  ±15.800 | 71.08  ±15.685 | 71.37  ±15.505 | 71.15  ±15.212 | 70.87  ±14.621 |
| ApoE ε4, n (%)* |  |  |  |  |  |  |
| Carriers | 368 (67) | 362 (67) | 365 (67) | 376 (69) | 391 (71) | 378 (68) |
| Noncarriers | 178 (32) | 178 (33) | 181 (33) | 167 (31) | 156 (29) | 176 (32) |

*****These overall data were previously published (20).

^†^Unpublished observation.

ApoE, apolipoprotein E; SD, standard deviation.

**Supplementary table 2. Cumulative dose (mg/kg) received, stratified by ApoE status, in the OTC population by March 20, 2019.**

|  | **EMERGE** | | | | | |
| --- | --- | --- | --- | --- | --- | --- |
|  | **Low dose** | | | **High dose** | | |
|  | **ApoE Ꜫ4+**  **3 mg/kg**  **(n = 7)** | **ApoE Ꜫ4-**  **6 mg/kg**  **(n = 2)** | **Total**  **(n = 9)** | **ApoE Ꜫ4+**  **10 mg/kg**  **(n = 9)** | **ApoE Ꜫ4-**  **10 mg/kg**  **(n = 4)** | **Total**  **(n = 13)** |
| **Cumulative dose (mg/kg) received**  Mean (SD) | 54.0  (2.65) | 44.0  (59.4) | 51.8 (21.58) | 115.3 (57.14) | 152.5 (15.00) | 126.8 (50.51) |
| **Number of 10 mg/kg doses received**  Mean (SD) | 0 | 0 | 0 | 9.3  (5.34) | 13.3  (1.50) | 10.5  (4.81) |
|  | **ENGAGE** | | | | | |
|  | **Low dose** | | | **High dose** | | |
|  | **ApoE Ꜫ4+**  **3 mg/kg**  **(n = 9)** | **ApoE Ꜫ4-**  **6 mg/kg**  **(n = 6)** | **Total**  **(n = 15)** | **ApoE Ꜫ4+**  **10 mg/kg**  **(n = 5)** | **ApoE Ꜫ4-**  **10 mg/kg**  **(n = 5)** | **Total**  **(n = 10)** |
| **Cumulative dose (mg/kg) received**  Mean (SD) | 55.7  (1.00) | 74.8 (39.88) | 63.3 (25.75) | 119.8 (28.27) | 119.0 (65.04) | 119.4 (47.28) |
| **Number of 10 mg/kg doses received**  Mean (SD) | 0 | 0 | 0 | 8.6  (3.91) | 10.2  (5.85) | 9.4  (4.77) |

**Supplemental Table 3. Summary of ARIA incidents of the Japanese safety MRI population.**

|  | EMERGE | | | ENGAGE | | |
| --- | --- | --- | --- | --- | --- | --- |
|  | **Placebo**  **(n=43)** | **Low dose**  **(n=37)** | **High dose**  **(n=40)** | **Placebo**  **(n=33)** | **Low dose**  **(n=29)** | **High dose**  **(n=38)** |
| ARIA-E, n (%) | 0 | 5 (13.5) | 8 (20.0) | 1 (3.0) | 2 (6.9) | 5 (13.2) |
| ApoE ε4 carriers, n/total (%) | 0 | 4/24 (16.7) | 5/25 (20.0) | 1/21 (4.8) | 1/22 (4.5) | 5/24 (20.8) |
| ApoE ε4 noncarriers, n/total (%) | 0 | 1/13 (7.7) | 3/15 (20.0) | 0 | 1/7 (14.3) | 0 |
| ARIA-H microhemorrhage, n (%) | 1 (2.3) | 5 (13.5) | 8 (20.0) | 3 (9.1) | 2 (6.9) | 5 (13.2) |
| ARIA-H macrohemorrhage, n (%) | 0 | 0 | 0 | 1 (3.0) | 0 | 1 (2.6) |
| ARIA-H superficial siderosis, n (%) | 0 | 2 (5.4) | 2 (5.0) | 0 | 1 (3.4) | 3 (7.9) |

Note: Any MRI events of brain microhemorrhage or localized superficial siderosis were reported as ARIA-H microhemorrhage or ARIA-H superficial siderosis, respectively. Only patients who were dosed and had ≥1 postbaseline safety MRI were included.

ApoE, apolipoprotein E, ARIA, amyloid related imaging abnormality; ARIA-E; ARIA-edema; ARIA-H, ARIA-microhemorrhages, macrohemorrhages, or superficial siderosis.

**Supplemental Figure 1. Study enrollment and timing of key protocol amendments for the overall population and the Japanese subgroup**


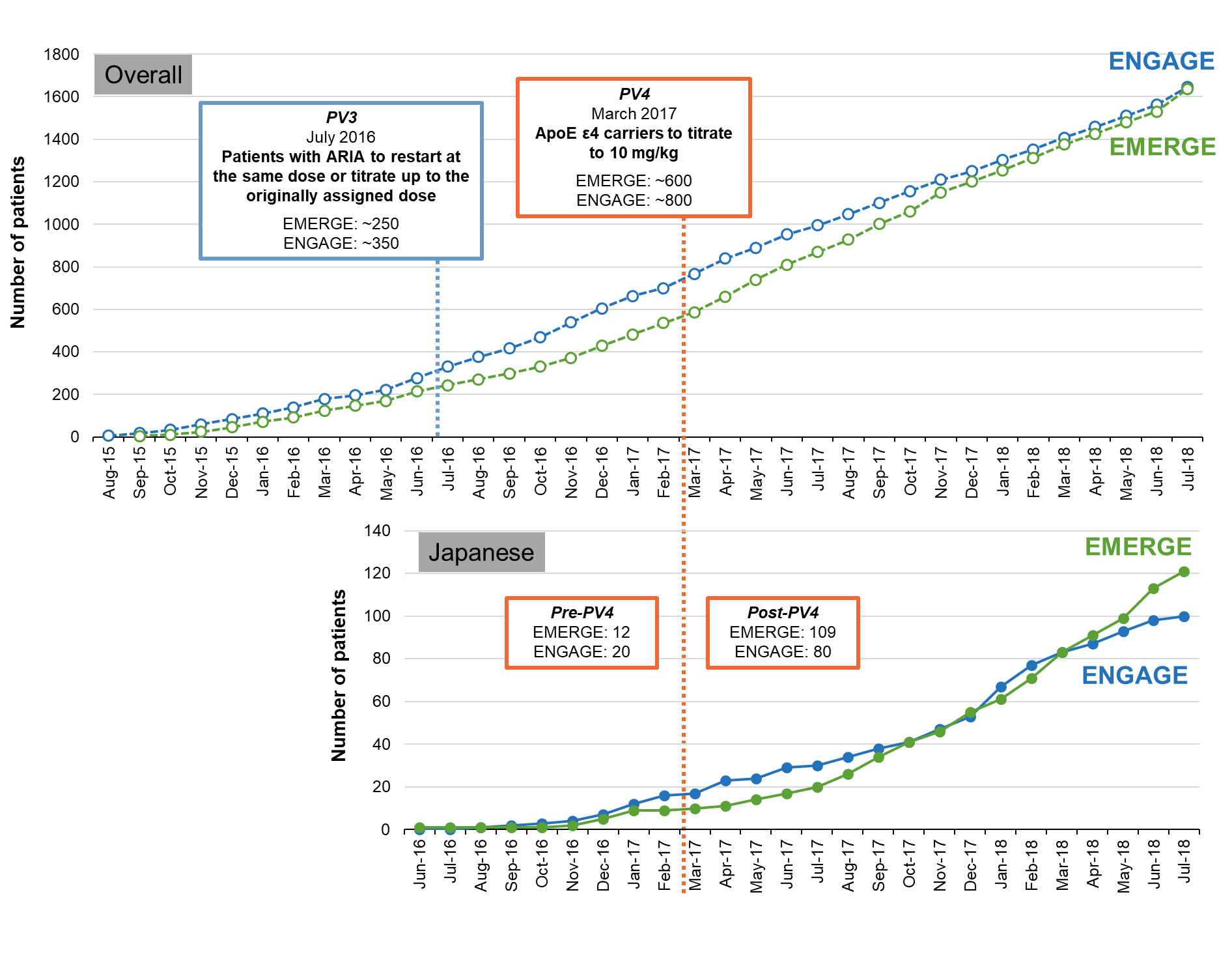


ApoE, apolipoprotein E; ARIA, amyloid related imaging abnormality; PV3, protocol version 3; PV4, protocol version 4.

**Supplemental Figure 2. Observed serum PK profiles in the Japanese and non-Japanese populations**

**
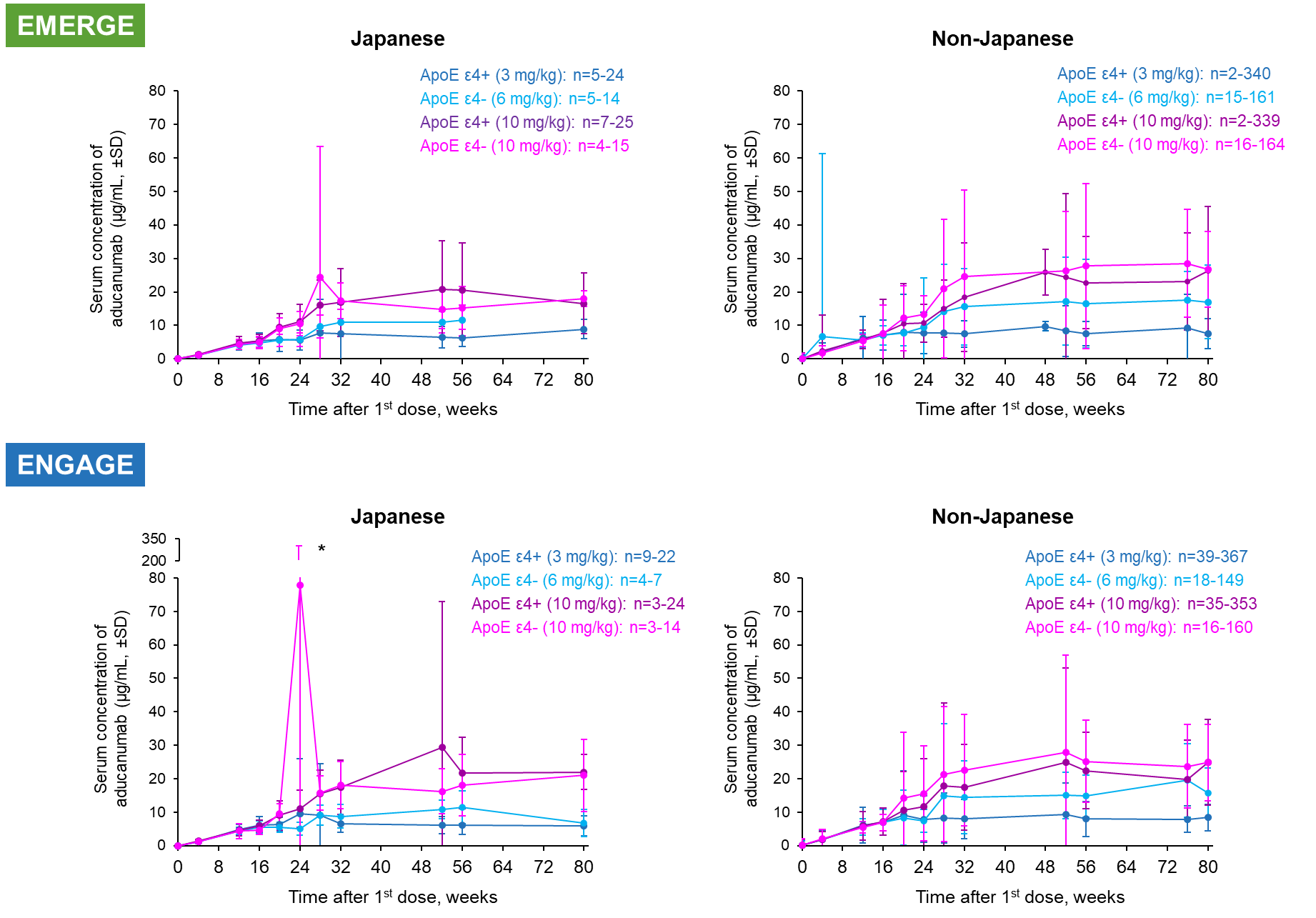
**

*One Japanese participant in ENGAGE had an increased serum aducanumab concentration value compared with the rest of the participants in the high-dose ApoE ε4– group (n=13) at Week 24
